# Tumor-infiltrating lymphocytes and tumor-associated macrophages in cancer immunoediting: a targetable therapeutic axis

**DOI:** 10.3389/fimmu.2025.1655176

**Published:** 2025-09-11

**Authors:** Sumon Mukherjee, Ahana Ghosh, Sourio Chakraborty, Udit Basak, Sumoyee Mukherjee, Tanya Das, Gaurisankar Sa

**Affiliations:** ^1^ Division of Molecular Medicine, Bose Institute, P-1/12, Calcutta Improvement Trust Scheme VII M, Kolkata, India; ^2^ Department of Biochemistry and Medical Biotechnology, Calcutta School of Tropical Medicine, Kolkata, India

**Keywords:** immunoediting, immunotherapy, TIL, TAM, tumor microenvironment

## Abstract

Immunotherapy has transformed the landscape of cancer treatment, offering hope to patients who were once considered beyond the reach of effective care. However, its success is restricted to a limited fraction of patients. This discrepancy in response is largely due to the complex and dynamic nature of the tumor immune-microenvironment. At the heart of this complexity is the concept of cancer immunoediting—a dynamic process through which the immune system both sculpts and is shaped by the tumor. This process unfolds in three key stages: Elimination, Equilibrium, and Escape, each representing a shifting balance between immune defenses and tumor adaptation. Central to this interaction are tumor-infiltrating lymphocytes (TILs) and tumor-associated macrophages (TAMs). TILs are frontline defenders in targeting tumor cells, while TAMs can either hinder or facilitate tumor growth based on their polarization. As cancer progresses, immune selection pressure induces phenotypic alterations that promote immune evasion, fostering an environment detrimental to effective immune response. This review explores the role of these immunological components in each phase of immunoediting and their impact on the efficacy or failure of immunotherapy. Gaining deeper insight into these interactions is crucial for developing advanced immunotherapies that reshape tumor microenvironment and expand the reach of immunotherapy to more patients.

## Introduction to cancer-immunoediting: overview and phases

**Figure 1:**
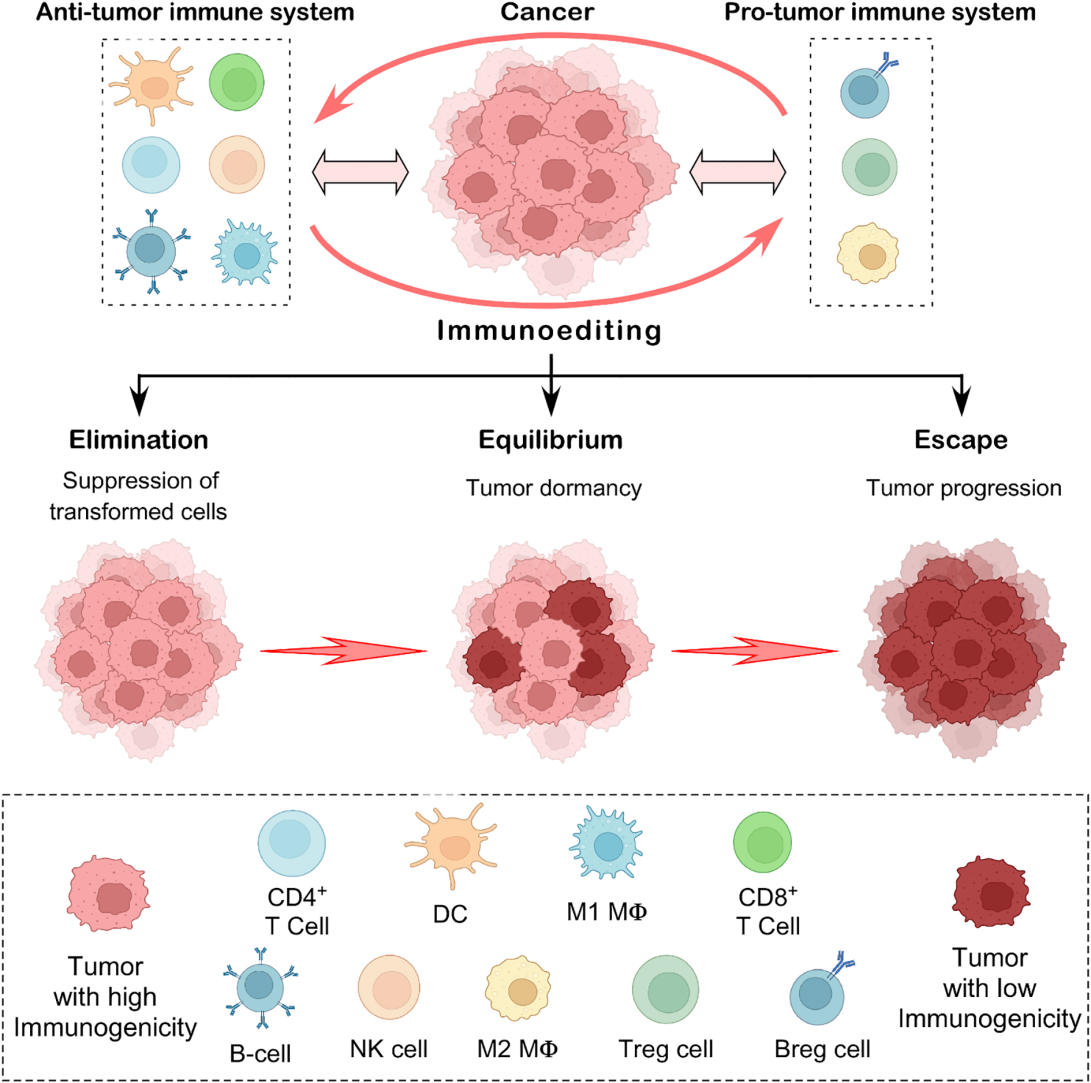
The concept of cancer immunoediting. This illustration depicts the three key phases of cancer immunoediting: elimination, equilibrium, and escape. In the elimination stage, anti-tumor immune effector cells act as defenders, identifying and destroying emerging transformed tumor cells. When some cancer cells evade this response, the process enters the equilibrium phase, where the immune system keeps the tumor in a dormant state but cannot eliminate it. Eventually, in the escape phase, resistant tumor cells adapt and grow unchecked, often spreading to other parts of the body. This concept captures the complex and evolving relationship between the immune system and cancer, reflecting its ability to both protect against and support tumor progression.

Tumor suppression has traditionally been thought of as a cell-intrinsic function driven by pathways involving proteins such as p53. When these signaling pathways fail, it leads to malignant transformations. On the other hand, the cancer immunosurveillance concept suggests that external factors, such as the immune system, also inhibit tumor growth (1). The crosstalk between immune cells and tumor cells is becoming an emerging subject in tumor immunology. Cancer immunoediting focuses on the immune system’s combined host-protective and tumor-sculpting functions. The immune system can simultaneously inhibit as well as promote tumor growth (2, 3). Robert Schreiber and his colleagues, using mouse tumor models, hypothesized that cancer immunosurveillance remains active in immunocompetent hosts during cancer progression (4). The authors further showed that by attempting to control cancer proliferation, the immune system is pushing the cancer cells to evolve into more resistant variants. This dual role of immunity to control cancer (elimination, equilibrium) and thereby promote it (escape) served as the basis for the theory of cancer immunoediting (3). The three E’s, elimination, equilibrium, and escape, are the three crucial stages that have been suggested as the major features of cancer immunoediting that progress from immune surveillance to immunological escape (**Figure 1**) (2, 3).

Under the fundamental principles of cancer immunoediting, it is hypothesized that in the elimination phase, the immune effector cells remove the newly transformed cells. If they are unable to completely eradicate the tumor, then the tumor cells enter the equilibrium phase (5). The tumor variants entering the equilibrium phase are characterized by reduced immunogenicity and higher resistance to immune response. They arise from immune selection and immune sculpting that they adopt to survive the elimination phase. Tumors eventually enlarge and employ several defense mechanisms to evade immune response within the tumor-microenvironment (3, 5). Studies in the mouse MCA sarcoma model provided the experimental evidence in favor of the cancer immunoediting theory. These investigations revealed the significance of several immune cell populations including T cells and natural killer (NK) cells and molecules (such as FASL, TRAIL, Perforin, and type-I and type-II IFNs) in the immunoediting of cancer (2, 6, 7). The idea of cancer immunoediting has evolved, changing our understanding of the interactions between the immune system and tumors. However, much more study is required to clarify the molecular and cellular dynamics of cancer immunoediting. This review discusses the fundamentals of immunoediting, specifically immune surveillance and escape, as well as the crucial role of immune effector cells, particularly tumor-infiltrating lymphocytes (TILs) and tumor-associated macrophages (TAMs), in this process. Gaining more knowledge about the processes by which immunoediting occurs during tumor growth could lead to new developments in cancer immunotherapy.

## Cancer-immunity cycle meets cancer immunoediting

**Figure 2:**
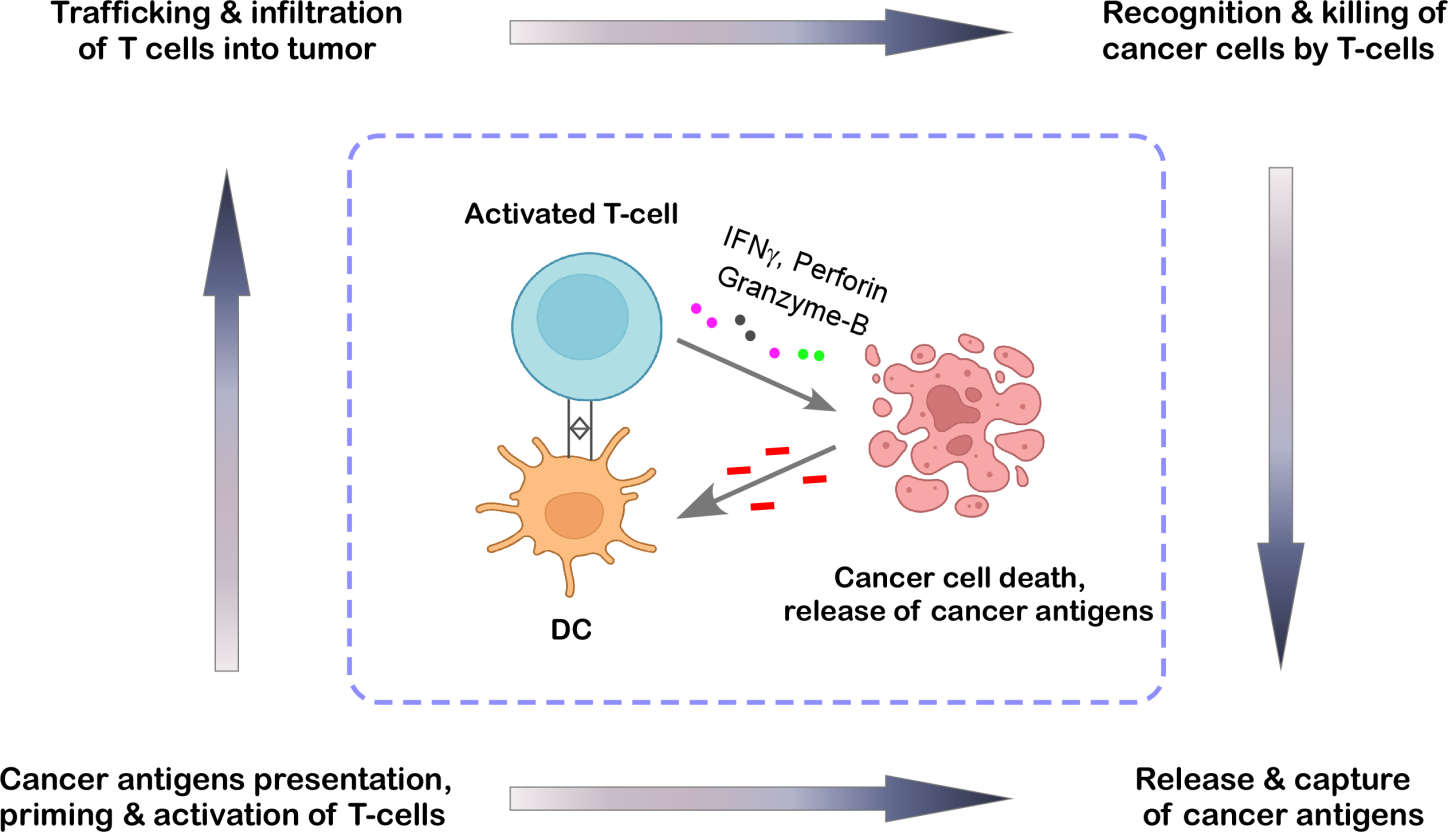
The cancer-immunity cycle. This cycle represents the stepwise process through which the immune system detects and restricts tumor growth. It begins with dendritic cells (DCs) capturing antigens released by cancer cells. These antigens are then presented to T cells, which become activated and proliferate. Once primed, the T cells travel to the tumor site, where they recognize and destroy cancer cells by releasing cytotoxic molecules, thereby reinforcing the immune response in a continuous loop. The illustration was created from Biorender.com.

An efficient anti-cancer immune response requires a sequence of events called the cancer-immunity cycle (**Figure 2**) (8, 9). To begin the process, the secreted tumor neoantigens are first taken up by antigen-presenting cells such as dendritic cells (DCs). DCs present the collected antigens to T cells on major histocompatibility complex (MHC)-I and MHC-II molecules. The next step involves priming and activating effector T cell responses against the specific antigens. At this point, the immune response strategy is clear, and the ratio of T-effector cells to T-regulatory cells, a crucial balance, determines the outcome. Following their trafficking to the tumor bed, the activated effector T cells kill their target cancer cell after selectively identifying and binding to cancer cells through the interaction of their T cell receptor (TCR) to the corresponding antigen coupled to MHC-I. Additional tumor-associated antigens are released when the tumor cell is killed, expanding the scope and depth of the response in later cycle revolutions (9). If this cycle becomes ineffective in the elimination stage of cancer immunoediting, it leads to immunosurveillance evasion and poor patient survival. Tumor-derived factors in the tumor microenvironment (TME) may suppress those effector cells by generating immunosuppressive immune cell types, hiding tumor antigens, influencing DCs and T cells to perceive antigens as self rather than foreign, thereby preventing effector responses; or preventing T cells from entering the tumor (9, 10). Thus, the cancer-immunity cycle is especially important at the initial stage of cancer immunoediting and entails the immune system’s identification and destruction of cancer cells. Here, we’ve covered the role that TILs and TAMs play in each stage of cancer immunoediting and tried to provide insight into how to apply this understanding to the development of future immunotherapies.

## Roles of immune cells in the phases of cancer immunoediting

### Elimination phase

In this initial stage of cancer immunoediting, many anti-tumor immune subsets, including T cells, B cells, innate lymphoid cells (ILCs), such as NK cells and NKT cells, and M1-type macrophages, destroy cancer cells by invading the tumor sites in response to molecular signals (11, 12).

### T cells

Since T cells are one of the largest subsets in the TME and primarily contribute to TILs, they have been the subject of extensive research (13). Both the antitumor response and the invasion of the TME depend on them. Numerous signaling molecules, including chemokines (e.g., CCL3, CCL4, CCL5, CCL20, CXCL9, CXCL10, CXCL11, and CXCL16) and their receptors, cause infiltration into the TME (14–17). In head and neck cancer, MHC-I molecules display antigens to TCRs, which in turn activate cytotoxic T lymphocytes to specifically attack tumor cells and trigger their apoptosis (18, 19).

**Figure 3:**
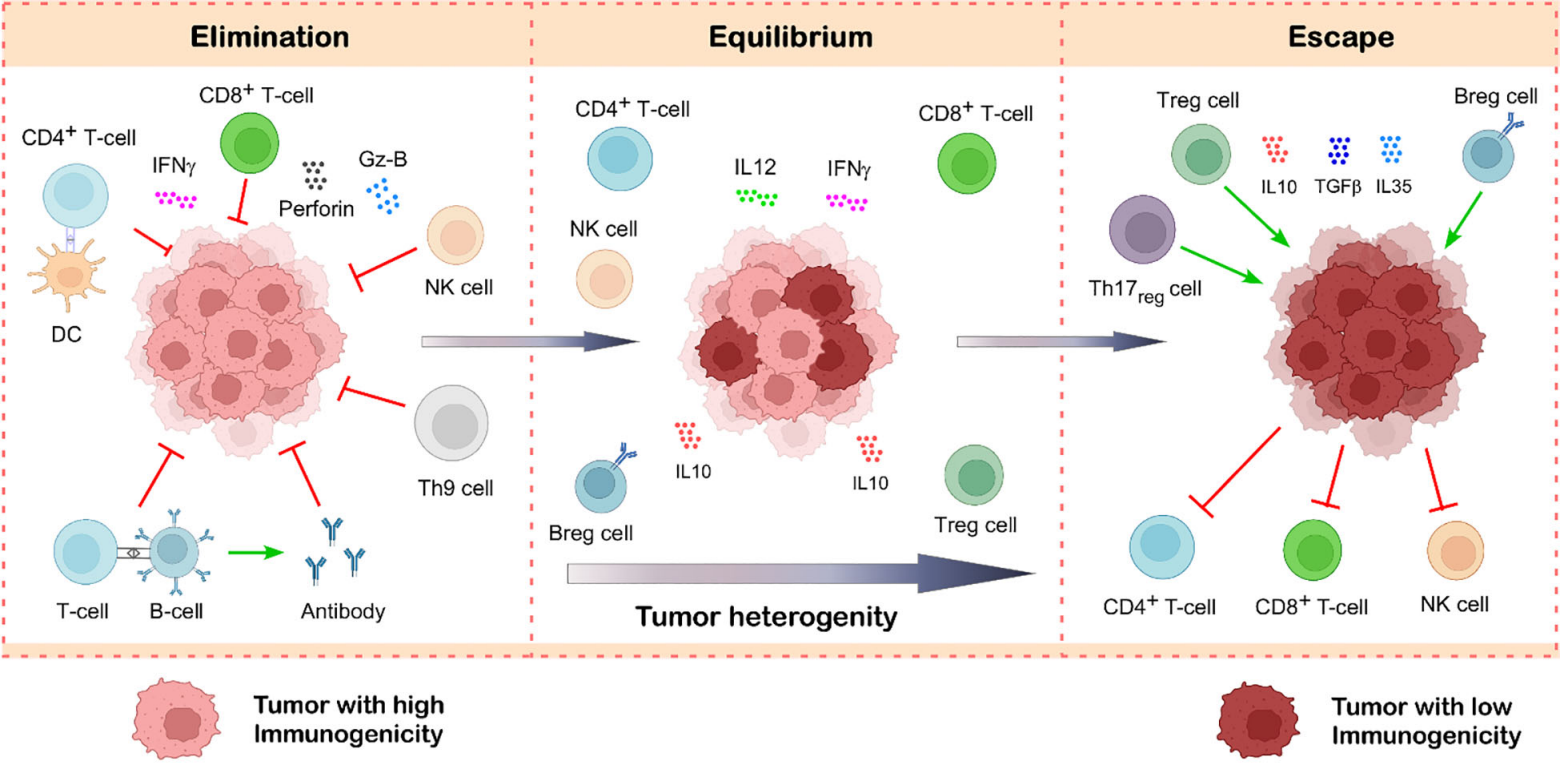
Tumor-infiltrating lymphocytes in cancer immunoediting. Cancer immunoediting unfolds in three interconnected stages: elimination, equilibrium, and escape. During the elimination phase, immune cells such as CD8^+^ T cells, NK cells, CD4^+^ T cells, B cells, and Th9 cells actively detect and destroy newly transformed tumor cells through various effector mechanisms. If a subset of tumor cells survives this immune attack, the process transitions into the equilibrium phase, where immune surveillance limits tumor expansion without fully eliminating it. Over time, immune pressure leads to the selection of tumor variants with reduced immunogenicity. Simultaneously, the tumor promotes the accumulation of immunosuppressive cells like Treg cells and Breg cells, which makes the tumor microenvironment immunosuppressive by suppressing the function of anti-tumor immune cells. This shift creates an immunosuppressive tumor microenvironment, marking the escape phase, wherein the tumor evades immune control, resumes growth, and metastasize. Illustration created with BioRender.com.

Death receptor ligation, FASL, TRAIL, and the release of granzyme B and perforin can all trigger apoptosis (**Figure 3**) (20). Naïve CD4^+^ T cells, when activated by tumor antigen-primed APC and exposed to various cytokines, can differentiate into different subsets, such as T-helper cells: Th1, Th2, Th9, Th17, Th22, and T-regulatory (Treg) cells (21, 22). Th1 cells secrete interleukin-2 (IL2) and interferon-γ (IFNγ) to aid in CTL activation. IFNγ plays a crucial role in inhibiting tumor growth, enhancing MHC expression, and limiting angiogenesis in cancers such as brain tumors, melanoma, and colon cancer (23–25). Th2 cells release cytokines like IL4 and IL13, which can induce eosinophil recruitment into the TME (26). CD4^+^ T cells, together with immune partners like DCs, B cells, and NK cells support cytotoxic T cells in carrying out their effector functions; (27–29). Evidence indicates that Th1 cell differentiation and function are closely related to those of other immune cells in the TME. For example, it has been demonstrated that DCs, M1 macrophages, and B cells promote Th1 cell development by generating cytokines such as IL12 and IFNγ (30–32).

According to a recent analysis, Th9 cells in the TME of solid tumors are linked to a strong anti-tumor immune response *via* both innate and adaptive immunological pathways (33). Th9 cells primarily rely on IL9 and IL21 to perform their anti-tumor function. CCR6^+^ DC and CCR6^+^ CD8^+^ T cells are drawn into the tumor bed and have a higher chance of surviving when IL9 stimulates epithelial lung cells to generate CCL20 (34). IL21 from Th9 cells stimulates CD8^+^ T cell proliferation and boosts NK cytolytic activity and IFNγ production (34). Recombinant IL9 activation increased the cytotoxicity of tumor-specific mice CD8^+^ T cells, whereas blocking IL9-signalling decreased the production of granzyme B and perforin in human CD8^+^ T cells (35). In a murine model, studies showed that a reduction in tumor-specific Th17 cells facilitated the advancement of B16 melanoma, with this effect directly associated with the production of IFNγ. Martin-Orozco et al. provide solid evidence that Th17 facilitates the activation of tumor-specific CD8^+^ T cells, hence indirectly inhibiting tumor growth (36, 37). Th17 cells possess the ability to acquire Th1-like characteristics and secrete substantial amounts of IFNγ, likely enhancing anticancer immune responses (38, 39). DCs release IL12, which promotes CD4^+^ T cells to develop into Th1 cells and triggers a strong Th1 immune response (40). Memory T cells may stay in pathogenic tissue, as in the case of resident memory T (TRM) cells, or they may circulate in the immune system to react quickly and strongly to subsequent exposure to antigens (41).

### Natural killer and natural killer T cells

Similar to CTLs, NK cells induce apoptosis in tumor cells by releasing cytolytic granules that include granzymes and perforins (**Figure 3**) (20). **Table 1** lists examples of activating and inhibitory receptors present on NK cells and NKT cells. Natural killer group-2 member-D (NKG2D), DNAX accessory molecule-1 (DNAM-1), NKp30, NKp44, and NKp46 are some of these activating receptors. Activating receptors use an immunoreceptor tyrosine-based activation motif (ITAM) located on their cytoplasmic tail to transduce signals. NKG2D is also expressed by NKT cells, CD8^+^ αβT cells, and γδ T cells (20, 54). As a result of signal transduction triggered by NKG2D receptor-ligand interaction, the tumor cells are degranulated by NK cells. While activating receptors initiate cytolytic activity, inhibiting receptors, such as those that detect MHC-I, limit their ability to kill normal, healthy cells (54, 55).

**Table 1:** Activating and inhibitory receptors expressed by NK and NKT cells.

Receptor	Cell	Ligand	Function	References
Activating receptors				
NKG2D	NK, NKT	MICA/B; ULBP1-6	Recognizes stress-induced ligands; triggers cytotoxicity & IFNγ production	(42) (43)
NCRs (NKp30, NKp44, NKp46)	NK	Viral HA, HSPGs, B7-H6 (NKp30)	Mediates tumor cell lysis; NKp30 also regulates DC crosstalk	(42) (43)
DNAM-1 (CD226)	NK, NKT	CD112 (PVRL2), CD155 (PVR)	Enhances cytotoxicity against tumor cells	(44) (43)
CD16 (FcγRIIIa)	NK	IgG (antibody Fc region)	Mediates ADCC (antibody-dependent cytotoxicity)	(42) (43)
2B4 (CD244)	NK, NKT	CD48 (on hematopoietic cells)	Dual role: Activates NK cells (when bound to SAP) or inhibits (without SAP)	(45) (43)
KIR2DS2 (Activating KIRs)	NK	HLA-C (subset)	Triggers cytotoxicity; role in anti-tumor responses	(46)
Inhibitory receptors				
KIR2DL/3DL	NK	HLA-A, -B, -C	Suppresses NK activation to prevent autoimmunity (“missing self” detection)	(47)
NKG2A (CD94/NKG2A)	NK, NKT	HLA-E (nonamer peptide)	Blocks NKG2D/DNAM-1 signaling; maintains self-tolerance	(42) (48)
PD-1	NKT, NK (exhausted)	PD-L1/PD-L2 (on tumors/APCs)	Induces exhaustion; dampens antitumor responses	(49) (50)
TIGIT	NK, NKT	CD155 (PVR), CD112 (PVRL2)	Competes with DNAM-1; suppresses IFN-γ & cytotoxicity	(42) (51)
TIM-3	NKT, NK	Galectin-9, HMGB1, CEACAM1	Induces exhaustion; decreased cytotoxic activity	(52) (53)

NKT cells are a subset that is characterized by their dual roles. They express αβ-TCR to identify endogenous and foreign lipid antigens presented on CD1d, a non-classical antigen-presenting protein resembling MHC class-I. On the other hand, they show NK cell characteristics due to the expression of CD56, CD16, and Fc receptor on their surface and granzyme production (15, 56). Leukocyte function-associated antigen-1 (LFA1) expression, CCR2, and CXCR6 can all mediate the recruitment of NKT cells (20, 56). NKT cells release Th1 and Th2 cytokines, including IFNγ, tumor necrosis factor-α (TNFα), IL2, IL4, IL5, IL6, IL10, IL13, IL17, IL21, transforming growth factor-β (TGFβ), and granulocyte monocyte-colony stimulating factor (GM-CSF), upon activation via αβ-TCR and CD1d interaction (57).

### B cells

B cells are vital for their antigen-presenting capability since B cell receptor (BCR) is far more specific to antigen recognition than the other APCs (58). In the TME, B cells act in multiple ways. They increase the density of T cells in the tumor by activating CD4^+^ T cells. They also mediate the conversion of CD4^+^ T cells and CD8^+^ T cells into distinct functional subsets (59). B cells can modulate the activity of other APCs, such as DCs and macrophages, enhancing their ability to present antigens effectively (58, 60, 61). A “helper” function for B cells in the tumor immune response is that activated CTLs can interact with soluble CD27 produced by CD19^+^ B cells, which promotes their survival and proliferation (62). The discovery that IgG2b-mediated activated B cells were highly lethal to tumor cells raised the possibility that B cells also contributed to the inhibition of tumor growth (63). Additionally, pulmonary host B cells were found to enhance IFNγ production and facilitate NK cell killing of tumor cells, suggesting that effector B cells may have a protective role against cancer (64, 65). CD20^+^ B cells recruit CD8^+^ T cells by the production of chemokines such as CCL3, CCL4, CCL5, CXCL10, and CXCL13. CXCL13 is a major chemokine that recruits B and T cells to malignancies (66–68). By stimulating T-cells, particularly CD4^+^ T cells, B cells improve the density and responsiveness of T cells in the TME (59).

### M1-tumor-associated macrophages

**Figure 4:**
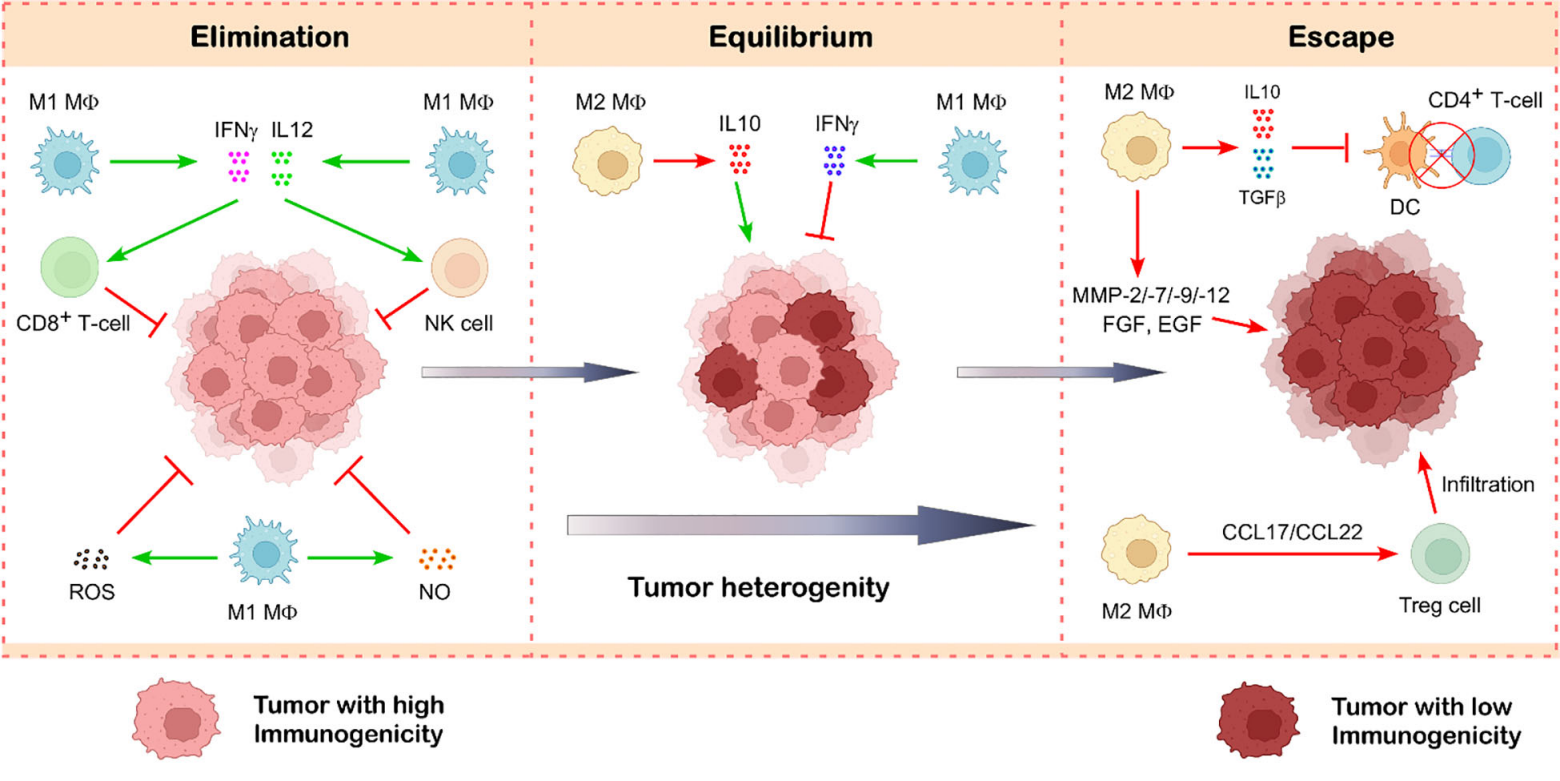
Role of tumor-associated macrophages in cancer immunoediting. Tumor-associated macrophages exist in two main functional states—M1 and M2—that play opposing roles in tumor progression. M1-like TAMs are typically anti-tumorigenic and contribute to tumor suppression by releasing cytotoxic molecules such as ROS, NO, IFNγ, and IL12, which help destroy cancer cells. In contrast, M2-like TAMs support tumor growth and progression. They secrete immunosuppressive cytokines like TGFβ and IL10, which dampen the anti-tumor immunity. Additionally, M2 macrophages produce growth factors and matrix-remodeling enzymes such as metalloproteinases, facilitating tumor growth and metastasis. These macrophages also promote the infiltration of Treg cells, further reinforcing an immunosuppressive tumor microenvironment. Illustration created with BioRender.com.

Type-1 macrophages (M1) have anti-tumor characteristics that allow them to distinguish between transformed and healthy cells. The way by which M1-type macrophages eliminate tumor cells after identifying them has been demonstrated to be impacted by two different factors (69). Using a variety of mechanisms, M1-type macrophages directly mediate cytotoxicity to kill tumor cells (70). One of the ways they achieve this is by generating molecules that kill tumors, such as ROS and NO, which have a cytotoxic effect on tumor cells (71) (**Figure 4**). The second pathway, known as antibody-dependent cell-mediated cytotoxicity (ADCC), kills tumor cells by selectively targeting them using anti-tumor antibodies (72). The destruction of tumor cells by NK cells is improved when Dectin1 is expressed on M1 macrophages (69). By secreting large amounts of pro-inflammatory cytokines IFNγ and IL12, which have anti-tumor activity, M1 macrophages incite NK cell and cytotoxic T cell infiltration and activation in the tumor site, indicating an indirect mechanism of stopping the spread of cancer (69, 73) (**Figure 4**).

### Equilibrium phase

The subsequent phase of cancer immunoediting, referred to as the equilibrium phase, occurs when tumor cells undergo continuous reshaping to increase their resistance to immune effector cells (74). Immune selection promotes tumor cells with lower immunogenicity as a result of this strategy. The paradox of tumor growth in an immunocompetent individual may be answered with the emergence of highly evolved resistant cancer cells (1, 2). Although these tumor cell variations are less immunogenic, random genetic changes inside tumor cells may result in more unstable malignancies. Nucleotide-excision repair instability (NIN), microsatellite instability (MIN), and chromosomal instability (CIN) are the three forms of genetic instability linked to cancer that have been suggested to be the cause of the “mutator phenotype” of tumor cells (74). Moreover, immune selection pressure increases the likelihood of tumor cell clones with a non-immunogenic phenotype (1, 3).

Of the three stages of cancer immunoediting, the equilibrium phase is probably the longest and may continue for many years (1, 74). It entails the constant removal of tumor cells and the emergence of resistant tumor variants as a result of immune selection pressure. It is unclear how exactly TILs and TAMs contribute to preserving equilibrium, even though their function in the elimination and escape phase has been thoroughly investigated. Research has shown that adaptive immunity components CD4^+^ T cells, CD8^+^ T cells, IL12, and IFNγ are in charge of keeping tumor cells in balance by exerting immune selection pressure (**Figure 3**) (1, 2). This Darwinian selection phase destroys many of the original tumor variants while tumor variants with distinct mutations that boost resistance to immune attack survive and proliferate to reach the next phase (1, 74). Moreover, the relative balance between effector and regulatory immune cells is also considered to be the most important factor in this phase (75). Additionally, studies showed that tumors remain in an equilibrium condition when IL12, which promotes elimination, and IL23, which promotes persistence, are balanced (76). Besides this, Th1 to Th2 cytokine bias also plays a very important role in maintaining the tumor in this phase (77, 78). By decreasing the activity of anti-tumor immune cells, anti-inflammatory cytokines such as IL10 and its expression level play a significant role in the equilibrium phase and cause the tumor to enter the escape phase (79).

### Escape phase

In the escape process, the surviving tumor variants that bypass the immune detection and elimination start to proliferate in an uncontrolled manner and employ various mechanisms to create an environment for their survival (80). In this phase, tumor-promoting immune cells like Treg cells, Breg cells, and M2-type TAMs infiltrate the tumor and promote tumor growth by suppressing the function of anti-tumor immune subsets (69, 81). In addition, Th1 to Th2 cytokine bias is also considered an important factor that maintains the tumor in an equilibrium phase. Immunosuppressive cytokines like IL10 and its expression level determine the shift from equilibrium to the escape phase.

### T cells

Tumor cells downregulate MHC class-I to evade CTL-mediated death by impairing their ability to process and deliver antigens (19). Additionally, tumor cells frequently exhibit dysregulated expression of death receptors like FAS and/or upregulate anti-apoptotic molecules like BCL2. TH2 cells secrete IL4, IL5, IL10, IL13, and IL17 - all of which have been shown to contribute to the tumor-promoting role of this subtype, even though IL4, IL5, and IL13 have been shown to contribute to the growth and metastasis of cancer (82, 83). CD4^+^ Th2 cells encourage the growth of tumors by suppressing Th1 cell-mediated immunity and boosting angiogenesis (84). However, recent literature has reported that IL10 has a dual pro- and anti-tumorigenic role (85, 86). IL10 elicits an anti-inflammatory immune response, downregulates Th1 cytokine function, and MHC class-II antigen presentation (87). At the same time, binding of IL10 with its cognate receptor activates signal-transducer and activator of transcription-3 (STAT3)-signaling and transcription of anti-apoptosis and cell cycle progression genes, further strengthening the pro-tumorigenic effect (88). Greater IL9^+^ cell infiltration in the tumor tissue was associated with worse prognosis, greater frequencies of Treg cells, and decreased cytotoxic capability of CD8^+^ T cells and NK cells in patients with muscle-invasive bladder cancer (89). The main issue with the Th9 function in the TME is that different tumor types exhibit inconsistent behavior.

One essential cytokine that controls the activity of mast cells and Treg cells is IL9. Feng et al. found that in B-cell non-Hodgkin’s lymphoma (NHL), IL9 was associated with immunosuppression mediated by CD117^+^ mast cells and FOXP3^+^ T-regulatory cells (90). Additionally, studies showed that Th9 cells enhanced the phosphorylation of STAT3, which has been linked to a poor prognosis for patients with HCC, hence increasing the production of CCL20. CCL20 is known to stimulate the migration and proliferation of tumor cells (34, 91). Th17 cells have two functions in the TME. This cell subgroup inhibits anti-cancer responses by producing immunosuppressive adenosine, and promotes angiogenesis as well as tumor development by releasing pro-inflammatory IL17A (92). The TME may encourage Th17 cells to differentiate into suppressive Treg cells, which would aid in immunosuppression (37, 93). Compared to the density of Th17 cells in the patient’s surrounding, non-tumor tissue, a noticeably higher number of Th17 cells infiltrate malignancies. This increased Th17 cell presence in tumor tissue is consistent across a wide spectrum of cancers, suggesting that tumors themselves generate components that facilitate Th17 cell trafficking to the site of illness (94). Th17, along with Th2, are involved in chronic inflammation, which encourages the growth and development of tumors (37, 84).

### Natural killer cells

To avoid immune surveillance by NK cells, tumor cells downregulate their surface ligands, thereby obstructing anti-tumor recognition. TGFβ, IFNγ, STAT3, hypoxia, proteolytic shedding, the generation of soluble ligands, and certain microRNAs collectively facilitate the downregulation of ligands (95–97). Cancer cells release immunosuppressive microvesicles, such as exosomes, that display NKG2D ligands (NKG2DLs) on their surface. These ligands bind to NKG2D receptors on NK cells and CD8^+^ T cells, leading to receptor downregulation, internalization, or degradation. This decoy interaction prevents NKG2D from recognizing tumor-expressed ligands, reducing tumor detection and impairing the anti-tumor activity of NK cells and CD8^+^ T cells (95, 98, 99).

### T-regulatory cells

T-regulatory cells, characterized by their immunosuppressive properties, infiltrate the TME *via* four distinct mechanisms. Initially, by transitioning to the TME from the circulatory or lymphatic systems. Signaling molecules such as chemokines and their receptors (e.g., CCL1-CCR8, CCL5-CCR5, CCL22-CCR4, CCL28-CCR10, and CXCL12-CXCR4) facilitate the ingress of Tregs into the TME (17, 100, 101). Secondly, immunosuppressive chemokines and cytokines can enhance their development in the TME. Third, by the expansion mediated by DC activation. Ultimately, effector T cell differentiation into Treg cells by TGFβ (102). CD25^+^ and FOXP3^lo^ Treg cell progenitors are the origins of thymically mature Tregs (101, 103). Tregs facilitate tumor growth and metastasis when they are recruited to the TME. Various suppressive functions of Treg cells are facilitated by immunosuppressive cytokines secreted by Tregs, such as TGFβ, IL10, and IL35 (**Figure 3**) (103). Treg cells can restrict the activity of antigen-presenting cells by downregulating the expression of CD80 and CD86 in a CTLA4-dependent way, therefore preventing the presentation of tumor antigens and the activation of tumor-specific T cells (104). Since an almost total suppression of CD8-mediated cytolytic activity is primarily reliant on TGFβ-signaling, and CD8^+^ T cells with a dominant negative TGFβ receptor were resistant to this suppression, and CD8^+^ T cells with a dominant negative TGFβ receptor were resistant to this suppression, TGFβ secretion from Treg cells can regulate CTL function and reduce anti-tumor immunity (105).

Previous findings indicate that Treg cells inhibit the proliferation and functionality of CD4^+^ and CD8^+^ T cells by depleting available IL2 and activating IL2/IL2-receptor signaling (101, 106). A recent study investigating the antigen specificity of Treg cells across several malignancies revealed that intra-tumoral Treg cells selectively responded to tumor antigens, leading to the activation and clonal expansion of Treg cells (107). Treg cells inhibit the interactions between antigen-presenting cells and T cells, hence obstructing the maturation and functionality of APCs, which subsequently impedes the activity of effector T cells (108, 109). Furthermore, Treg cells diminish the efficacy of NK cells. Treg cells can suppress anti-tumor immunity by diminishing responses from both the innate and adaptive immune systems (110, 111). T-regulatory cells are associated with a low survival probability in cancer patients (112, 113).

### B-regulatory cells

Numerous studies indicate that Breg cells infiltrate human malignancies and suppress anti-tumor immune responses through the expression of immune checkpoint molecules (PDL1) and immunosuppressive cytokines (IL10, IL35, and TGFβ) (**Figure 3**) (114, 115). Additionally, a significant presence of Breg cells was observed to diminish antibody-mediated humoral immunity in cancer patients (115). The effector molecules of Breg cells, such as TGFβ, IL10, and IL35, can diminish effector T cell responses and/or promote Treg differentiation, thereby resulting in enhanced tumor progression (116). Breg cells impede the development of naïve T cells into Th1 and Th17 (117). They also exert analogous effects on dendritic cells and macrophages (117). Advanced hepatocellular carcinoma, gastric carcinoma, and prostate carcinoma often exhibit elevated levels of Breg cells, indicating that Breg cells may influence tumor development and metastasis (118–120). Breg cells secrete cytokines such as TGFβ, which can convert M1 macrophages into an M2 immunosuppressive phenotype and induce the differentiation of naive CD4^+^ T cells into Treg cells, leading to remodeling of the TME (121, 122).

### M2-tumor-associated macrophages

The growth of the tumor is closely associated with macrophage infiltration. Multiple studies have shown that tumor-associated macrophages can secrete various cytokines, such as platelet-derived growth factor (PDGF), TGFβ1, hepatocyte growth factor (HGF), epidermal growth factor (EGF) family, and basic fibroblast growth factor (BFGF), that facilitate tumor cell proliferation and survival (123). Another study indicates that the malignant invasion of phyllodes tumors is facilitated by a positive feedback loop of CCL5 and CCL18 between TAM and myofibroblasts. CCL5 triggers the AKT-signaling upon binding to its receptor to attract and repolarize tumor-associated macrophages. Consequently, TAMs secrete CCL18, which promotes the invasion of malignant tumors by converting mesenchymal fibroblasts into myofibroblasts (124). M2 macrophages can enhance the migration of tumor and stromal cells by secreting matrix metalloproteinases (MMPs), cathepsins, serine proteases, and various collagen types, along with other extracellular matrix components, which degrade the endothelial cell matrix membrane to facilitate neo-angiogenesis (70). The secreted factors promote epithelial-mesenchymal transition (EMT), resulting in tumor spread (125). In addition to synthesizing angiogenic factors, macrophages can express many enzymes that regulate angiogenesis, including MMP-2, cyclooxygenase-2, MMP-7, MMP-9, and MMP-12 (**Figure 4**) (70, 126). M2 macrophages secrete immunosuppressive cytokines that promote the Th2 response, whereas M1 macrophages elicit the Th1 response through the production of pro-inflammatory cytokines (127). TAM-derived TGFβ has been shown to induce HIF1/TRIB3-signaling, hence facilitating the advancement of cancer (128).

Tumor-associated macrophages produce large amounts of the immunosuppressive cytokine IL10, which inhibits the cytotoxic activity of Th1 cells, NK cells, and CD8^+^ T cells against tumor cells (129). This constrains the TME’s capacity to inhibit tumor growth. The recruitment of Tregs to the TME through the chemokine receptor CCR4 is significantly enhanced by tumor-associated macrophage-derived CCL17/CCL22 (17) (**Figure 4**). Reports indicate TAM-mediated Treg recruitment in liver, nasopharyngeal, and ovarian malignancies. These Tregs suppress anti-cancer CTL activity (130). M2-TAMs preserve their immunosuppressive characteristics and effectively deplete the anti-tumoral immune response by exhibiting elevated synthesis of immune checkpoint inhibitor (ICI) PDL1 and cytotoxic T-lymphocyte antigen-4 (CTLA4) ligand (69). The TGFβ and IL10 generated by TAM hinder the maturation and proliferation of DCs. Consequently, antigen presentation diminishes, leading to a compromised adaptive immune response (69, 131) (**Figure 4**).

## Interaction between tumor-infiltrating lymphocytes and tumor-associated macrophages

**Figure 5:**
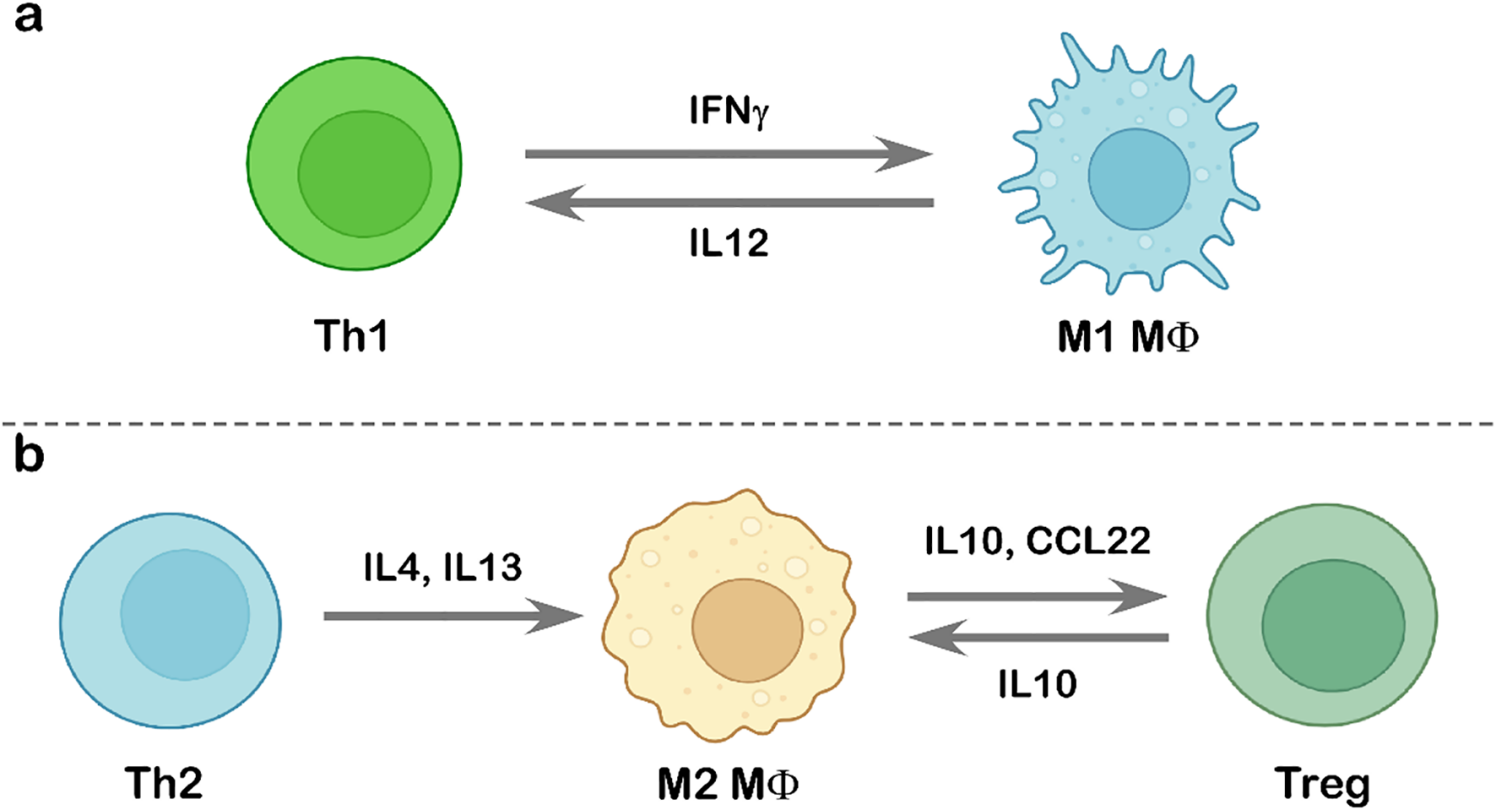
Interaction between tumor-infiltrating lymphocytes and tumor-associated macrophages. Schematic representation of the interplay between TILs and TAMs. **(a)** Interaction between Th1 cells and M1-polarized macrophages, highlighting the cytokine-mediated enhancement of anti-tumor immunity. **(b)** Th2 cell-induced M2 polarization through IL4 and IL13 signaling (left) and the subsequent interaction between M2-like macrophages and Treg cells (right). Illustration created using BioRender.com.

The crosstalk between tumor-infiltrating lymphocytes and tumor-associated macrophages plays a pivotal role in shaping the TME and influencing cancer progression. IFNγ secreted by Th1 cells can reprogram TAMs toward an M1 phenotype by activating the JAK–STAT1 signaling pathway, thereby enhancing anti-tumor immune responses (132, 133). M1 macrophages, in turn, produce cytokines and chemokines such as IL12, CXCL9, and CXCL10, which promote the polarization and recruitment of Th1 cells, strengthening type-1 immune responses (31) (**Figure 5**). In contrast, Th2-derived cytokines, including IL4 and IL13, drive M2 macrophage polarization. IL4, upon receptor binding, activates JAK, leading to STAT6 activation, a key regulator of M2 differentiation, which also induces peroxisome proliferator-activated receptor γ (PPARγ) to control the transcription of M2-specific genes (31, 132). In glioblastoma, the TME-derived glycoprotein chitinase-3-like protein 1 (CHI3L1), in association with Gal3, promotes M2 polarization, resulting in reduced CD4^+^ and CD8^+^ T cell populations (134). M2-TAM–secreted factors such as IL10, TGFβ, and CCL22 facilitate Treg cell expansion and recruitment into tumors (31, 135) (**Figure 5**). Additionally, MARCO^+^ macrophages suppress CD8^+^ T cell activity by promoting Treg proliferation (136), while M2-TAM–expressed ARG1 depletes L-arginine from the microenvironment, limiting T cell proliferation (137). B cell-derived IL10 has also been shown to contribute to M2 polarization in a melanoma model (31, 138), and in colon cancer, B cell–derived γ-aminobutyric acid (GABA) enhances IL10^+^ macrophage populations, which in turn inhibit CD8^+^ T cell function (139).

## Immunotherapy to counter immunoescape mechanisms

In the escape phase, tumor and stromal cells proliferate in a manner that evades the immune system. This generates a tumor-microenvironment that makes the immune system less effective. The objectives of immunotherapies are to restore the balance between the immune system and tumor cells, inhibit tumor growth, and eradicate tumor cells (140).

## Therapeutic approaches for tumor-infiltrating lymphocytes

Therapeutic strategies targeting TILs have evolved with the development of a novel adoptive cell therapy (ACT) approach known as TIL therapy, which harnesses the patient’s own immune system to fight cancer. In this method, immune cells, primarily T cells, are extracted from a patient’s tumor, expanded in the laboratory, and reinfused to boost the body’s ability to recognize and eliminate cancer cells. In February 2024, the U.S. Food and Drug Administration (FDA) approved Lifileucel, an autologous TIL-based therapy (141). A phase-II clinical trial (NCT03645928) is currently evaluating Lifileucel in combination with immune checkpoint inhibitors in patients with solid tumors (142). Recent advances have also focused on generating PD1–deficient TILs using CRISPR-Cas9 gene editing, which has shown enhanced anti-tumor efficacy. For instance, IOV-4001, a genetically engineered PD1 knockout TIL, is being investigated in a Phase I/II trial (IOV-GM1-201) for advanced non-small-cell lung cancer and unresectable or metastatic melanoma (141). Multiple TIL-based ACT trials (NCT01174121, NCT05417750, and NCT06488950) are actively recruiting for solid tumor studies (ClinicalTrials.gov). Another candidate, AGX148, a CD8^+^ TIL product expressing CD39 and CD103, is under Phase-I evaluation (NCT05902520) for advanced solid tumors, both as monotherapy and in combination with siRNA-mediated PD1 silencing (PH-762) (143). TBio-4101, a neoantigen-targeted TIL therapy designed using peptides with tumor-specific mutations to enrich tumor-reactive TILs, is being tested as a monotherapy in melanoma (NCT05628883) and alongside pembrolizumab in solid tumors (NCT05576077) (143).

**Figure 6:**
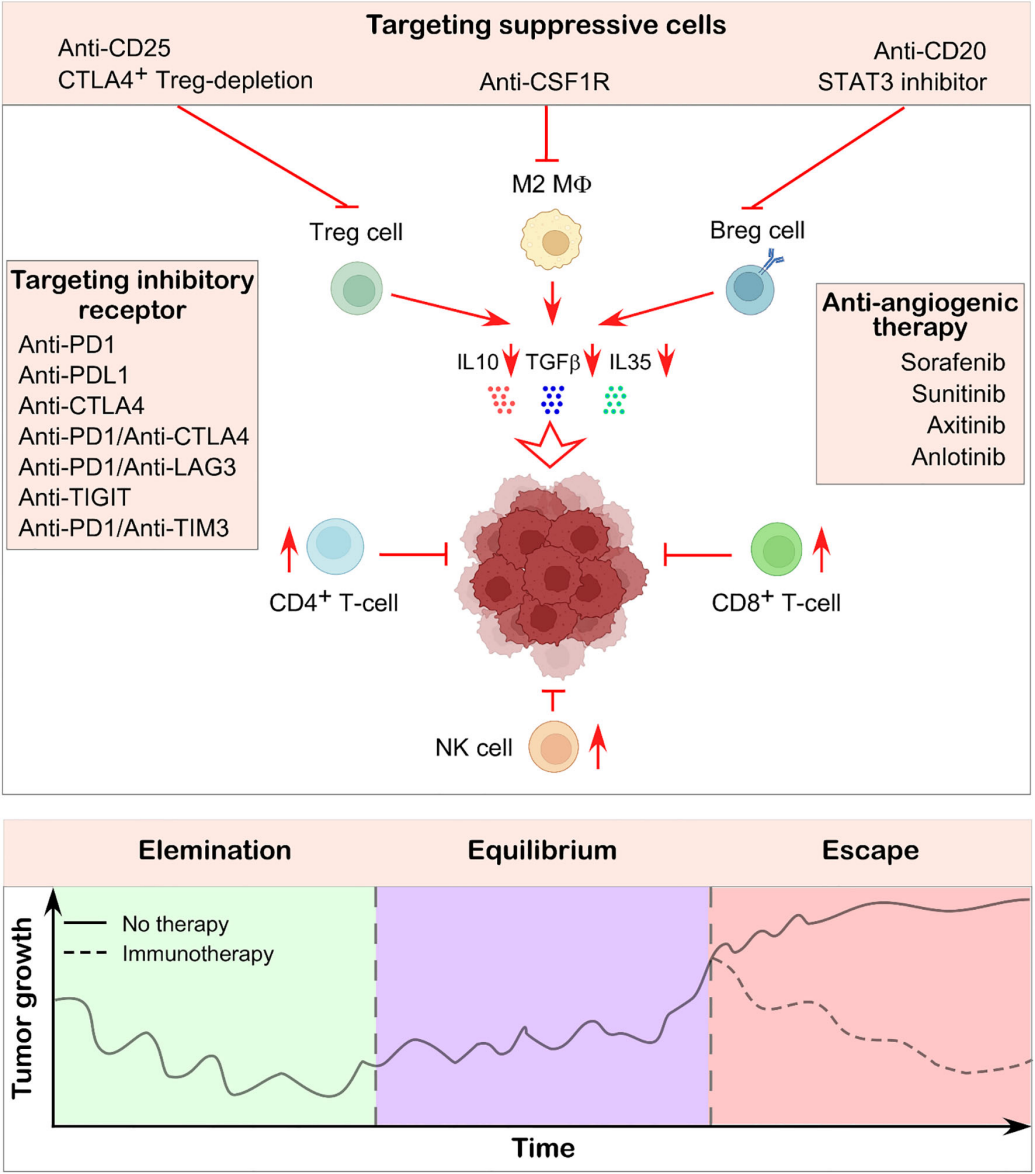
Immunotherapeutic strategy to suppress tumor growth. Immunotherapy aims to reinvigorate the body’s naturally hard-wired immune system to recognize and eliminate cancer cells. One approach involves using specific antibodies to target immunosuppressive cells within the tumor-microenvironment, thereby restoring the activity of effector immune cells and promoting anti-tumor responses. Another widely used strategy targets inhibitory immune checkpoints such as PD1, CTLA4, LAG3, PDL1 etc., which are often expressed on both immune cells and tumor cells. Blocking these molecules has been shown to enhance immune-mediated tumor destruction. Additionally, anti-angiogenic therapies contribute by normalizing the tumor microenvironment, improving immune cell infiltration, and further restricting tumor progression. The illustration in the lower panel shows how tumor growth rates alter depending on whether immunotherapy was used to counteract the immunosuppressive environment and restore immune balance. Illustration created using BioRender.com.

Targeting inhibitory immune receptors may serve as a therapeutic strategy for restoring immune normalcy. To develop new therapeutic strategies, numerous innovative immune checkpoint inhibitors, such as Lymphocyte Activation Gene 3 (LAG3), T cell immunoreceptor with Ig and ITIM domains (TIGIT), V-domain immunoglobulin suppressor of T cell activation (VISTA), and T cell immunoglobulin and mucin-domain containing-3 (TIM3), are undergoing extensive studies alongside PD1, PDL1, and CTLA4. Enhancing the proliferative and anti-tumor capabilities of effector T cells can be accomplished by targeting the elevated expression of LAG3 on CD8^+^ and CD4^+^ T cells in the bone marrow and blood of patients with multiple myeloma (144). Moreover, it has been shown that the combination of anti-LAG3 and anti-PD1 monoclonal antibodies enhances IFNγ production and T cell cytotoxicity, leading to more effective tumor growth inhibition (**Figure 6**) (145). Blocking TIGIT has been demonstrated to reverse NK cell depletion and enhance CD8^+^ T cell activity (146, 147). The co-expression of TIM3 and PD1 significantly affects T cell exhaustion and the loss of stemness (148). Unlike single-agent therapy, the simultaneous inhibition of TIM3 and PD1 has been shown to reinstate effector T cell functionality, induce more significant tumor shrinkage, and amplify the anti-tumor immune response (**Figure 6**) (149). In addition, CAR-T therapy has demonstrated considerable advantages in cancer treatment. It entails the genetic modification of T lymphocytes to express chimeric antigen receptors, so allowing them to specifically target tumor cells. Clinical trials indicated that CAR-T therapy exhibits a 45% efficacy rate in the treatment of multiple myeloma (150).

In a phase I/II clinical trial (NCT03056339), patients with recurrent or refractory B-cell lymphoma received cord blood-derived, CD19-targeted CAR-NK therapy, resulting in an overall response rate in 21 patients, 13 achieving complete responses and 8 partial responses. Among 49 treated patients, 18 demonstrated objective responses, including 14 complete and 4 partial responses (151). Another phase-I trial (NCT03383978) is evaluating the safety and tolerability of HER2-targeted NK-92 cells in combination with the immune checkpoint inhibitor ezabenlimab in patients with recurrent HER2-positive glioblastoma. In the United States, a phase-II study (NCT04847466) is underway to assess the efficacy of irradiated PDL1 CAR-NK cells combined with pembrolizumab and N-803 for recurrent or metastatic head and neck and gastric cancers. Collectively, these studies highlight the potential of CAR-NK cell therapies, particularly when paired with anticancer drugs, as a promising strategy for future cancer treatment.

It is crucial to target several immunosuppressive cells present in the TME to reshape the TME. Neutralizing antibodies against CD25 effectively eradicated CD25^+^ Treg cells in tumor-bearing mice, resulting in an enhanced infiltration of CD8^+^ T cells in the tumor (**Figure 6**) (152, 153). Furthermore, a mouse model has shown that the targeted elimination of Ctla4^+^ Treg cells results in complete tumor remission (154, 155). In contrast, the application of anti-CD20 monoclonal antibodies to eradicate Breg cells has yielded unsatisfactory outcomes for solid tumors, although it has demonstrated significant therapeutic efficacy in hematologic malignancies (156). Conversely, STAT3 inhibitors have been shown to reduce the synthesis of immunosuppressive cytokines and the proliferation of Breg cells (**Figure 6**) (157, 158). B-cell epitope–based vaccines represent an innovative direction in B-cell immunotherapy. For instance, a vaccine targeting the HER-2/neu peptide, an established tumor antigen, is being used in breast and ovarian cancers (159). Additionally, a phase I/II clinical trial (NCT04416984) is currently evaluating the safety and effectiveness of ALLO-501A, an allogeneic anti-CD19 CAR T cell therapy, alongside ALLO-647, an anti-CD52 monoclonal antibody, in patients with relapsed or refractory large B-cell lymphoma. Aside from this, current studies reveal that anti-angiogenic therapy enhances the immune-microenvironment and tumor vasculature. Anti-angiogenic therapy has led to the development of several strategies that target multi-targeted tyrosine kinases, including sorafenib, sunitinib, axitinib, and anlotinib (**Figure 6**) (160). Research has shown that neutralizing antibodies targeting VEGFA can effectively enhance CD8^+^ T cell activity and inhibit endothelial cell expression of FAS ligand, hence promoting effector T cell infiltration (161, 162). Anlotinib, a small-molecule tyrosine kinase inhibitor, was demonstrated to reduce PDL1 expression on endothelial cells, hence enhancing the equilibrium of immune cells within the tumor (163). **Table 2** also summarizes TIL-targeting therapies currently listed on ClinicalTrials.gov.

**Table 2:** Therapeutic approaches for tumor-infiltrating lymphocytes.

Target	Agents/drugs	Phase	Cancer	NCT ID
TIL	Lifileucel (LN-144) Lifileucel (LN-145)	II II	Metastatic melanoma metastatic non-small-cell lung cancer	NCT02360579 NCT05640193 NCT04614103
	Lifileucel, pembrolizumab	III		NCT05727904
	Fludarabine Aldesleukin Pembrolizumab	II	Breast, colorectal, ovarian, pancreatic tumor	NCT01174121
TIL and PD1	Lifileucel and ICIs in combination or single	II	Melanoma, Head and neck, NSCLC	NCT03645928
	Nivolumab	I	Non-Small Cell Lung Cancer	NCT03215810
Soluble LAG-3 protein and PD1	Eftilagimod alpha with pembrolizumab	II	Metastatic NSCLC and HNSCC	NCT03625323
PDL1, CTLA4	MEDI4736 with Tremelimumab	I	Advance solid tumor	NCT02261220
LAG-3	Sym022	I	Advance solid tumor	NCT03489369
PD1 and TIGIT	Atezolizumab Tiragolumab	I/II	Metastatic malignancy	NCT05394337
PD1	Toripalimab	I	Malignant lymphoma	NCT03316144
CTLA4	Ipilimumab	III	High-risk stage-III melanoma	NCT00636168
NK cell	CCCR-modified NK92 cell	I	NSCLC	NCT03656705
NK cell, CTLA4	BMS-986015 (anti-KIR) ipilimumab	I	Advance solid tumor	NCT01750580
NK cell, HER2 on cancer cells	Trastuzumab with NK	I/II	Breast cancer	NCT02843126
NK cell, B cell	NK cell with rituximab	I/II	B cell lymphoma	NCT02843061
CD40 agonistic mAbs	APX005M	I	NSCLC, melanoma, head and neck cancer	NCT02482168

## Therapeutic approaches for tumor-associated macrophages

One promising strategy to combat cancer involves reprogramming M2-type TAMs into the pro-inflammatory, anti-tumor M1 subtype. This repolarization can be stimulated through activation of toll-like receptors (TLR) on macrophages. Preclinical studies have shown that TLR7 agonists like imiquimod and 852A exhibit notable anticancer effects with durable therapeutic benefits (164, 165). Similarly, the TLR9 agonist lefitolimod has been evaluated in multiple clinical trials, including NCT02668770, where it promotes M1 polarization of TAMs and enhances anti-tumor immune responses (69, 166).

Targeting signal regulatory protein-α (SIRPα) or CD47 to restore the phagocytosis activity of TAM is another immunotherapeutic approach being used to treat different cancers. It is now feasible to successfully suppress tumor cell growth and metastasis by inhibiting CD47-SIRPα signaling, restoring macrophage phagocytosis of tumor cells, and enhancing the effector CD8^+^ T cell response (167). The versatile drug RRx-001, currently being investigated in clinical trials (NCT02518958), has shown the ability to promote the M1 phenotype in TAM. It also blocks the interaction between SIRPα on macrophages and CD47 on cancer cells, enhancing immune response against tumors (168, 169). Additionally, molecules like microRNA miR-340 and the polypeptide PEP-20 have been found to target CD47, boosting macrophage phagocytosis of cancer cells (170, 171). Macrophages expressing the scavenger receptor MARCO, which contributes to their transformation into immunosuppressive TAMs, display a pro-tumor, anti-inflammatory phenotype. Studies have demonstrated that targeting MARCO with specific antibodies can suppress TAM activation, shift macrophages toward the M1 phenotype, and enhance the effectiveness of anti-tumor immunotherapies in breast, colon cancer, and melanoma models (172, 173).

Macrophage colony stimulating factor-1 (CSF1)-CSF1R plays a critical role in TAM recruitment and survival, hence blocking this route could be a possible TAM treatment method. Study shown that the specific targeting of the CSF1R significantly inhibits tumor growth in mice with EL4 tumor and mouse mammary tumor virus transgenic mammary tumor model by depleting M2-type macrophages, indicating a promising target for cancer immunotherapy (**Figure 6**) (174). A randomized phase-II clinical trial involving patients with advanced triple-negative breast cancer found that combining the CSF1-targeting monoclonal antibody lacnotuzumab (NCT02435680) with chemotherapy led to better outcomes compared to chemotherapy alone (175). Bisphosphonates have also been reported to reduce the recruitment and infiltration of TAMs while promoting their apoptosis (69). Emactuzumab, another monoclonal antibody against CSF1, enhances anti-tumor immune responses by decreasing the number of F4/80^+^ TAMs within tumors and increasing the CD8^+^/CD4^+^ T cell ratio through inhibition of the CSF1/CSF1R-signaling pathway (164). When combined with paclitaxel, emactuzumab effectively suppresses the growth of advanced solid tumors and significantly reduces CSF1R^+^ TAM populations (176). Additionally, blocking the CCL2/CCR2 axis lowers invasive TAM numbers in tumors and enhances the effectiveness of combined immunotherapy and chemoradiotherapy (164, 177).

In glioblastoma animal models, inhibitors targeting the CCR2/CCL2 axis have been shown to significantly reduce M2-type TAM infiltration at tumor sites, leading to improved survival outcomes (69). In a clinical trial (NCT01413022) involving patients with locally advanced pancreatic cancer, the CCR2 inhibitor PF-04136309 was found to be safe and well tolerated when combined with chemotherapy (178). Suppressing TAM activity by blocking the CCL2/CCR2–STAT3 pathway using siRNA or pharmacological agents enhances macrophage phagocytosis and inhibits tumor growth and metastasis (164, 179). The CXCL12/CXCR4-signaling pathway also plays a key role in macrophage recruitment, and its inhibition alongside standard treatments has demonstrated anti-tumor effects (180). For example, a phase-I clinical trial (NCT02737072) combining durvalumab with the CXCR4 antagonist LY2510924 showed a favorable safety profile and promising responses in patients with advanced, treatment-resistant solid tumors (181). Additionally, in preclinical studies, macrophages engineered to express chimeric antigen receptors (CAR-M) markedly slowed tumor progression and extended survival (182, 183). Building on this, a clinical trial (NCT04660929) is investigating CAR-M therapies targeting HER2 for tumors that overexpress this receptor (184). **Table 3** provides a comprehensive overview of therapies currently approved or under active investigation that aim to target TAMs, based on information from ClinicalTrials.gov.

**Table 3:** Therapeutic approaches for tumor-associated macrophages.

Target	Agents/drugs	Phase	Cancer	NCT ID
CD47-SIRPa pathway	Lemzoparlimab	I	Multiple myeloma	NCT04895410
	Magrolimab	II I	Multiple myeloma Malignant Brain tumor	NCT04892446 NCT05169944
	RRx-001	I	Solid tumor, Lymphoma	NCT02518958
	Evorpacept	I	Urothelial carcinoma	NCT05524545
	CC-95251	I	Acute Myeloid Leukemia	NCT05168202
TLR9	Tilsotolimod	I II	Solid Tumor Malignant melanoma	NCT04196283 NCT04126876
TLR3	Poly-ICLC	I	Hepatocellular carcinoma	NCT05281926
	Rintatolimod	II	Prostate adenocarcinoma	NCT03899987
CD40-CD40L	CDX-1140	II	Ovarian clear cell adenocarcinoma	NCT05231122
	Mitazalimab	I/II	Metastatic pancreatic ductal adenocarcinoma	NCT04888312
	Selicrelumab	I/II I/II	Triple-negative breast cancer Pancreatic adenoma carcinoma	NCT03424005 NCT03193190
CSF1-CSF1R	Chiauranib	II III	Triple-negative breast cancer Small cell lung cancer	NCT05336721 NCT04830813
	TPX-0022	I/II	metastatic solid tumor	NCT03993873
	CM082	II	Small cell lung cancer II	NCT03904719
CCL2-CCR5	BMS-813160	I/II	Pancreatic ductal Adenocarcinoma	NCT03767582
	Carlumab	I	Solid tumor	NCT01204996

## Therapeutic approaches targeting tumor-infiltrating lymphocytes and tumor-associated macrophages

Emerging therapeutic strategies increasingly focus on simultaneously targeting tumor-infiltrating lymphocytes and tumor-associated macrophages to reshape the tumor immune microenvironment and boost treatment efficacy. In lung cancer, tumor-derived IL37 promotes the generation of MARCO-positive TAMs, which contribute to an immunosuppressive environment. Targeting MARCO or the IL37 receptor on macrophages has been shown to repolarize TAMs toward a more pro-inflammatory state, enhancing the cytolytic function of both natural killer cells and T cells while reducing Treg cell activity (136). Inhibition of the PI3Kγ pathway represents another promising avenue, as it leads to TAM repolarization with increased production of IL12 and IFNγ, alongside greater recruitment and maturation of CD8^+^ T cells within tumors (185, 186). Building on these findings, a phase-II randomized clinical trial (NCT03980041) is evaluating the combination of the PI3Kγ inhibitor IPI-549 with anti-PD1 immune checkpoint inhibitors in patients with advanced urothelial cancer.

Preclinical studies in mouse models of hepatocellular and liver cancer demonstrate that CCR2 antagonists reduce TAM populations and increase infiltration of CD8^+^ T cells, further promoting anti-tumor immunity (187, 188). Likewise, stimulator of interferon genes (STING) agonists have been shown to reprogram TAMs into the pro-inflammatory M1 phenotype and enhance CD8^+^ T cell infiltration in murine colorectal cancer models (189). A first-in-human phase-I trial (NCT03843359) is currently investigating GSK3745417, a STING agonist, combined with dostarlimab (anti-PD1) to assess safety, tolerability, and preliminary efficacy in patients with advanced solid tumors. Targeting molecular interactions within the tumor-microenvironment also shows promise. For example, disrupting the interaction between CHI3L1 and Galectin-3 (Gal3) using GMP, a Gal3-binding peptide mimetic, inhibits M2-TAM polarization and supports T cell proliferation in brain tumors (134). Similarly, combining anti-MS4A4A antibodies with PD1 blockade improves immune checkpoint inhibition by preventing macrophage M2 polarization and enhancing CD8^+^ T cell infiltration in advanced colorectal cancer (190).

In colon cancer, the GABA-A receptor antagonist picrotoxin significantly reduces IL10–secreting macrophages and restores effector T cell activity, further illustrating the potential of modulating TAMs to boost anti-tumor immunity (139). Ongoing clinical research includes a phase-I trial (NCT06637306) testing dupilumab, an IL4 receptor blocker, in combination with ICIs to repolarize M2 TAMs in breast cancer patients (191). Together, these approaches demonstrate the growing recognition that coordinated targeting of both TILs and TAMs holds substantial potential to remodel the immune landscape within tumors and improve therapeutic outcomes across a range of cancers.

## Factors determining immunotherapy outcomes

### Biomarkers

A key element influencing the success of immunotherapy lies within the TME, particularly the presence and characteristics of tumor-infiltrating lymphocytes and tumor-associated macrophages. Biomarkers associated with these immune cells serve as important predictors of treatment response. For instance, a high density of TILs, especially CD8^+^ T cells, has consistently been linked to improved clinical outcomes across multiple cancer types (192). Recent clinical analyses reveal that elevated levels of intratumoral tissue-resident memory T cells (TRMs), characterized by CD103^+^ and CD69^+^ expression, correlate with better responses to immune checkpoint inhibitor therapies in non-small cell lung cancer and oral cancer (193).

Markers indicative of T cell exhaustion, including TIM3, CTLA4, and PD1, reflect impaired T cell function and significantly impact the effectiveness of checkpoint blockade treatments (193). In melanoma patients treated with anti-PD1 therapy, the proportion of precursor exhausted T cells (TPEX cells) has been associated with longer-lasting responses, underscoring their potential as predictive biomarkers (193, 194). Furthermore, the presence of tertiary lymphoid structures (TLS), organized aggregates of B cells, DCs, and T cells, has been linked to enhanced therapeutic responses in ICIs-treated soft tissue sarcoma and melanoma patients (195, 196). Conversely, elevated FOXP3 expression, a marker for Treg cells, is often associated with immunotherapy resistance, immunosuppression, and poorer prognoses (197).

On the macrophage front, molecules expressed by different TAM subsets also play a critical role in shaping immunotherapy outcomes. M1 macrophages, known for their pro-inflammatory and anti-tumor functions, typically express markers such as CD80, CD86, and IL12. In contrast, M2 macrophages, which tend to support tumor progression and immune suppression, are characterized by markers including CD163, CD204, CD206, and IL10 (198). The ratio of M1 to M2 macrophages within the tumor has been linked to both patient survival and response to immunotherapy in cancers such as breast cancer (199). Additionally, high expression levels of TAM infiltration markers like CD68 and CD163 have been correlated with poor prognosis and treatment resistance in cervical and oral squamous cell carcinomas (200, 201). Together, these biomarkers provide valuable insights into the immune landscape of tumors and help guide personalized immunotherapy approaches for improved patient outcomes.

In the foreseeable future, immune checkpoint inhibitor therapy will predominate in cancer treatment (202–204). Neglecting immunophenotyping prior to the initiation of checkpoint inhibitor therapy may accelerate tumor progression (205). As a result, immunophenotyping contributes significantly to cancer research by revealing the immune landscape of tumors and supporting the design of targeted and personalized treatment strategies for diverse immunophenotypes, ultimately enhancing the efficacy of checkpoint inhibitor treatments.

### Tumor-infiltrating lymphocytes and tumor-associated macrophages heterogeneity

Immunotherapy outcomes can vary widely even among patients diagnosed with the same type of cancer, and one of the key factors influencing this variability is the composition of tumor-infiltrating immune cells within the TME. Tumors are often classified as “hot” or “cold” based on their immune cell infiltration profiles. “Hot tumors” are characterized by a TME rich in tumor-infiltrating lymphocytes, particularly CD8^+^ T cells, while “cold tumors” display lower levels of these cytotoxic immune cells (206). It is generally accepted that immunotherapies, such as immune checkpoint inhibitors, tend to be more effective in “hot tumors,” where CD8^+^ T cells play a crucial role in mounting an anti-tumor immune response (206, 207).

This section highlights how the heterogeneity of TILs and TAMs correlates with disease progression across various cancers. A recent study found that tumors with higher infiltration of CD8^+^ T cells and NK cells, combined with lower levels of M2-polarized TAMs, were significantly associated with improved progression free survival (PFS), indicated by hazard ratios (HR) less than 1, which reflects better PFS (208). For example, patients with adrenocortical carcinoma (ACC), liver hepatocellular carcinoma (LIHC), cervical squamous cell carcinoma and endocervical adenocarcinoma (CESC) exhibited lower M2 macrophage abundance and correspondingly better PFS (HR < 1). In contrast, cancers such as lower-grade glioma (LGG) and glioblastoma (GBM) demonstrated reduced median levels of CD8^+^ T cells and NK cells, alongside elevated M2 macrophage presence, correlating with poorer PFS (HR > 1) (208). The study also revealed notable differences within breast cancer subtypes: basal-like and HER2-enriched tumors showed significantly higher numbers of CTLs and a substantially lower likelihood of disease progression compared to luminal-A and luminal-B subtypes. Additionally, increased Treg cell infiltration was specifically associated with worse outcomes in renal clear cell carcinoma (208).

Together, these findings emphasize the complexity and heterogeneity of immune cell populations within the TME and underscore their critical influence on immunotherapy effectiveness and cancer progression. Understanding these nuances is essential for tailoring personalized treatment strategies and improving clinical outcomes.

### Heterogeneity of immune infiltration

Within a single tumor, the immune landscape can vary significantly across different regions, which greatly influences how the tumor responds to immunotherapy. Some areas may be rich in immune infiltration, characterized by the presence of activated T cells and dendritic cells, making these regions more susceptible to treatments like immune checkpoint blockade. Conversely, other parts of the TME may exhibit immunosuppressive features, marked by increased levels of myeloid-derived suppressor cells (MDSCs), Treg cells, Breg cells, and TAMs. These immunosuppressive cells dampen the activation and function of effector immune cells, leading to a diminished therapeutic response (209).

### Immune-related adverse events

Immune-related adverse events (irAEs) are commonly observed in patients undergoing treatment with immune checkpoint inhibitors and can significantly impact patient outcomes and the overall success of immunotherapy. ICIs work by blocking inhibitory immune checkpoints, thereby enhancing T cell–mediated immune responses against tumors. However, this disruption of immune regulation can lead to a loss of immune tolerance, causing autoreactive T cells to attack healthy tissues, a process that results in irAEs resembling autoimmune diseases (210). Additionally, ICIs may increase levels of pre-existing autoantibodies, such as antithyroid antibodies, which contribute to these immune complications (211). Overproduction of inflammatory cytokines in patients receiving ICIs is also associated with systemic toxicities (212).

Among the most common irAEs are gastrointestinal issues, with symptoms generally more frequent and severe in patients treated with anti-CTLA4 therapies compared to those receiving PD1 or PDL1 inhibitors (213). Clinical trials report that ICI-associated hepatitis occurs in approximately 2% to 15% of patients (214). Inflammatory arthritis affects about 1% of patients, while arthralgia is reported in 3% to 7%. Unlike dermatologic toxicities, which often appear early, other irAEs, such as arthritis, tend to develop later, typically around two months after starting PD1/PDL1 inhibitors and about one month following initiation of anti-CTLA4 therapy (212). Understanding and managing these immune-related side effects are critical to optimizing immunotherapy safety and effectiveness.

### Acquired resistance to therapy

Acquired resistance to immunotherapy often arises from disruptions in the tumor’s ability to present antigens effectively to T cells. Mutations affecting MHC molecules or components of the antigen-processing machinery can impair antigen presentation, preventing T cells from recognizing and attacking cancer cells. Such mechanisms have been documented in cancers like metastatic melanoma and prostate cancer (215).

Tumor metabolic changes also contribute to resistance. Cancer cells frequently undergo metabolic reprogramming, producing high levels of lactic acid through glycolysis. This acidifies the TME, which in turn suppresses T cell metabolism and impairs their function (215). Moreover, the tumor’s consumption of glucose can limit its availability to T cells, reducing mTOR signaling, IFNγ production, and the glycolytic capacity of T cells, further dampening their anti-tumor activity (216).

Another factor involved in acquired resistance is the increased production of adenosine by tumor cells, often driven by elevated expression of CD38. Adenosine inhibits T cell proliferation and cytotoxic functions, contributing to immune evasion (217). Genetic alterations can also play a role; for example, melanoma patients who develop resistance to PD1 blockade have been found to carry loss-of-function mutations in JAK1 or JAK2 genes. These mutations render tumor cells less responsive to the antiproliferative effects of T cell–derived IFNγ, allowing cancer cells to escape immune attack (218).

Lastly, tumor cells may undergo “antigenic drift,” where changes in tumor epitopes alter their antigenicity. This shift enables cancer cells to evade recognition by T cells, facilitating immune escape and therapy resistance (219). Together, these mechanisms highlight the complexity of acquired resistance and underscore the need for strategies that can overcome or prevent these barriers to effective immunotherapy.

## Concluding remarks

In conclusion, comprehending cancer through the lens of immunoediting provides valuable insight into the dynamic and often paradoxical relationship between the immune system and tumor cells. By focusing on the regulatory roles of tumor-infiltrating lymphocytes and tumor-associated macrophages, we highlight the importance of decoding the immune landscape within the tumor-microenvironment. Restoring and enhancing the immune system’s inherent defenses requires a deep grasp of how these cellular components interact under both healthy and pathological conditions. As immunotherapy continues to evolve, identifying the reasons for therapeutic resistance remains a critical step toward improving outcomes.

Resistance remains a significant challenge in cancer treatment, as tumor cells employ diverse strategies to escape immune detection. These include modifying immune evasion pathways, increasing the expression of alternative immune checkpoints, and losing tumor antigens that are targets for immune cells. However, several promising research directions are emerging. Advances in personalized medicine now allow for the development of tailored therapies that consider each patient’s unique immune landscape and tumor characteristics, potentially improving treatment effectiveness. Additionally, growing evidence highlights the important role of the gut microbiota in shaping responses to cancer immunotherapy, making modulation of the gut microbiome a compelling approach to enhance therapeutic outcomes.

With growing access to advanced immune profiling and the integration of artificial intelligence, we are poised to develop more precise and personalized immunotherapeutic strategies. Ultimately, these advancements possess the capacity to transform immunotherapy into a widely effective and enduring remedy in the battle against cancer.

## Glossary

APC: Antigen Presenting Cell
Breg: B-regulatory cell
CCL: Chemokine (C-C motif) ligand
CAR-T: Chimeric Antigen Receptor – T cell
CD: Cluster of Differentiation
CSF1: Colony stimulating factor-1
CTL: Cytotoxic T lymphocyte
CTLA4: Cytotoxic T Lymphocyte Associated Protein – 4
CXCL: Chemokine (C-X-C motif) ligand
CXCR: Chemokine (C-X-C motif) receptor
DC: Dendritic cell
FASL: FAS Ligand
ICI: Immune Checkpoint Inhibitor
IFN: Interferon
IL: Interleukin
LAG-3: Lymphocyte Activation Gene 3
MHC: Major Histocompatibility Complex
MMP: Matrix Metalloproteinase
NK: Natural Killer
NKG2D: Natural Killer Group 2 Member D
NKT: Natural Killer T cells
NO: Nitric Oxide
PD1: Programmed Death receptor 1
PDL-1: Programmed Death Ligand 1
STAT3: Signal Transducer and Activator of Transcription 3
TAM: tumor-associated macrophage
TCR: T cell receptor
TGFβ: Transforming Growth Factor β
Th: T helper cell
TIGIT: T cell immunoreceptor with Ig and ITIM domains
TIL: Tumor Infiltrating Lymphocyte
Tim-3: T cell immunoglobulin and mucin-domain containing – 3
TLR: Toll-like receptor
TME: Tumor microenvironment
TRAIL: TNF-related apoptosis-inducing ligand
Treg: T-regulatory cell
